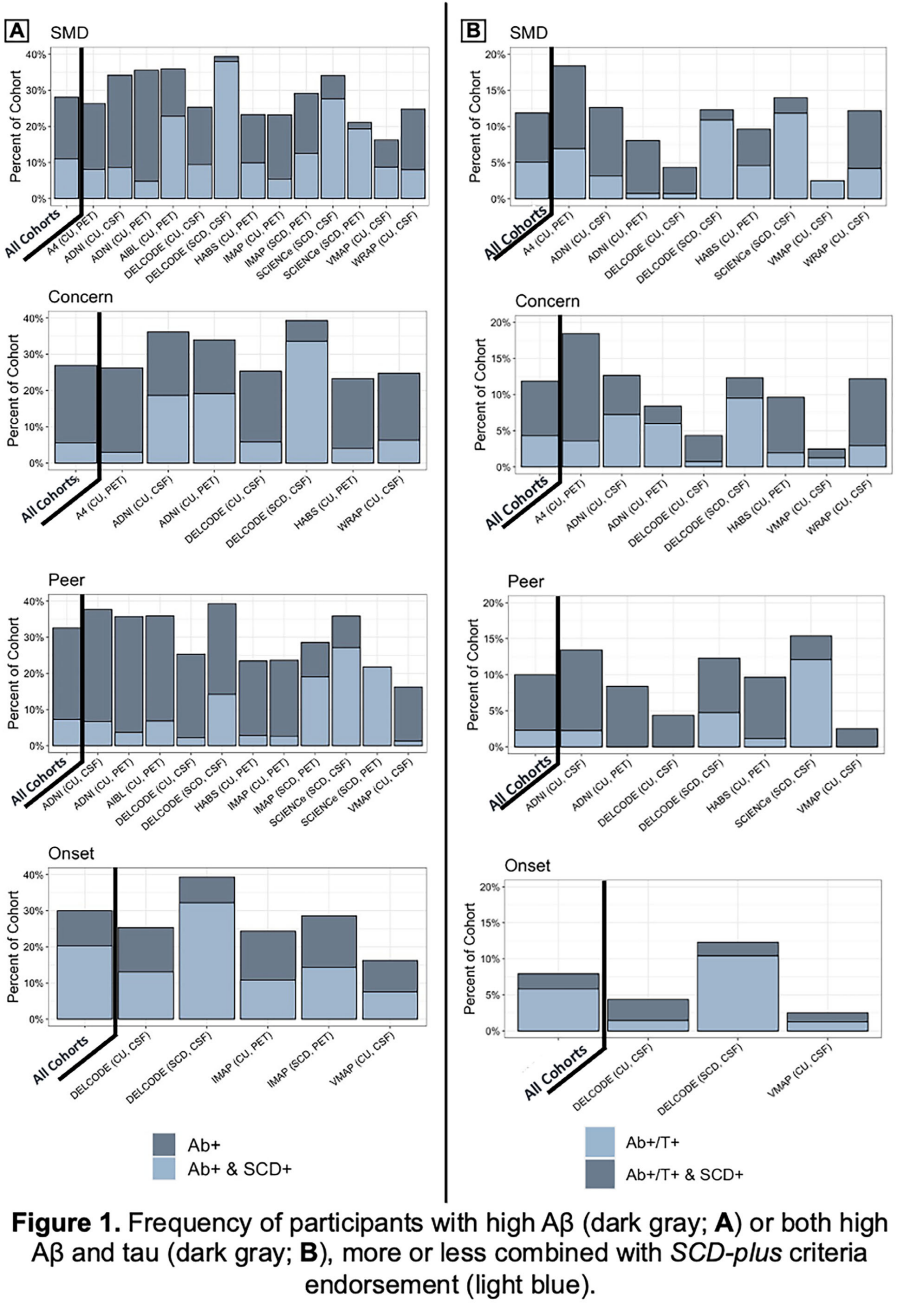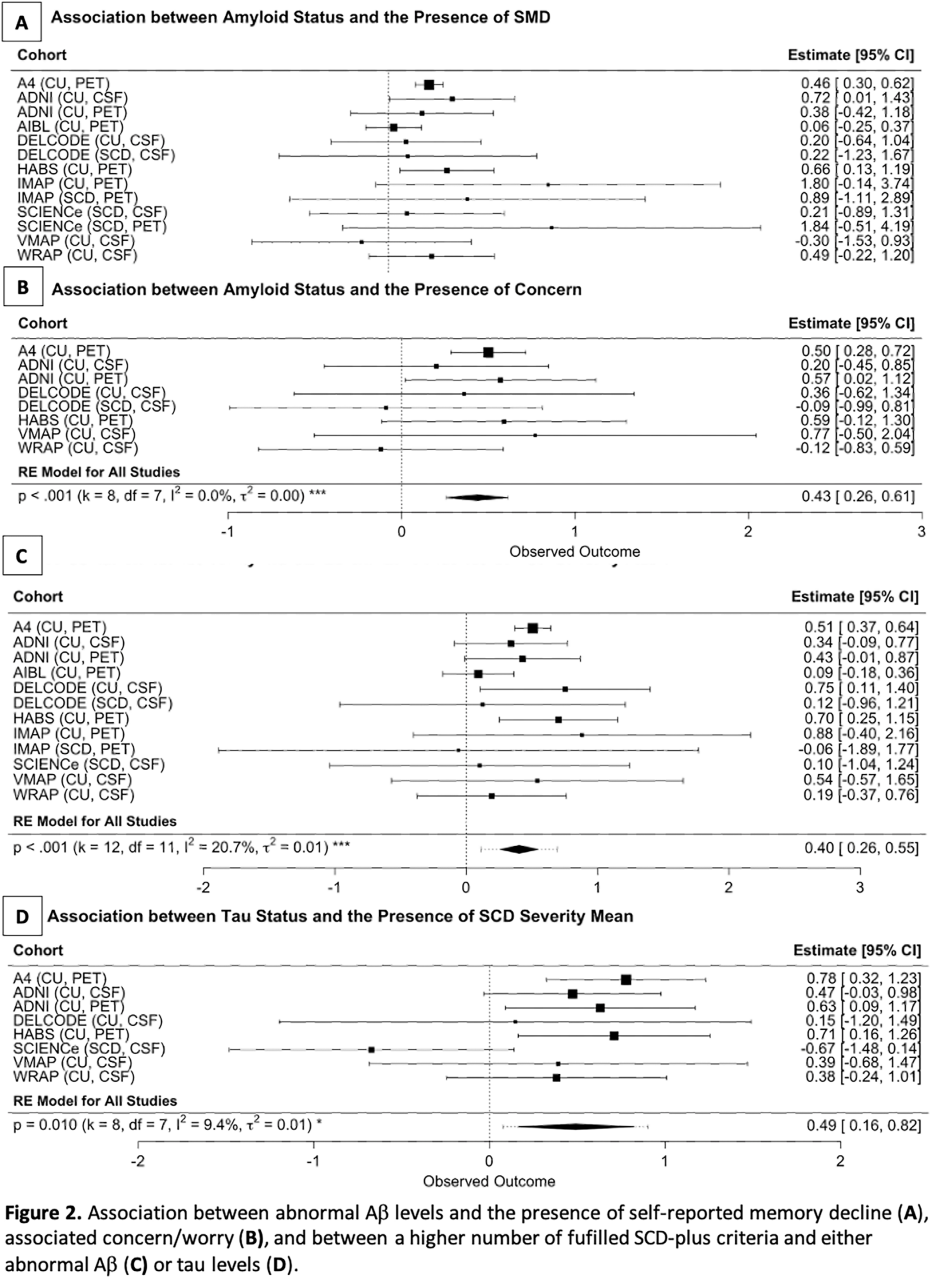# Association between self‐reported SCD‐*plus* criteria and Alzheimer’s disease biomarkers in cognitively unimpaired older adults: meta‐analyses

**DOI:** 10.1002/alz.088662

**Published:** 2025-01-03

**Authors:** Elizabeth Kuhn, Hannah M Klinger, Rebecca E Amariglio, Frank Jessen, Michael Wagner, Gael Chételat, Dorene M Rentz, Reisa A Sperling, Jarith L. Ebenau, Elke Butterbrod, Wiesje M. van der Flier, Sietske A.M Sikkes, Lorena Rami, Gonzalo Sánchez‐Benavides, Katherine A. Gifford, Carol A. Van Hulle, Rachel F Buckley, the Alzheimer’s Disease Neuroimaging Initiativethe Australian Imaging Biomarkers and Lifestyle flagship study of ageingthe A4 study team, the DELCODE study groupthe Harvard Aging Brain Study

**Affiliations:** ^1^ German Center for Neurodegenerative Diseases (DZNE), Bonn Germany; ^2^ Department of Neurodegenerative Diseases and Geriatric Psychiatry, University of Bonn Medical Center, Bonn Germany; ^3^ Massachusetts General Hospital, Boston, MA USA; ^4^ Center for Alzheimer Research and Treatment, Brigham and Women’s Hospital, Harvard Medical School, Boston, MA USA; ^5^ Department of Psychiatry, University of Cologne, Medical Faculty, Kerpener Strasse 62, Cologne Germany; ^6^ Excellence Cluster on Cellular Stress Responses in Aging‐Associated Diseases (CECAD), University of Cologne, Cologne Germany; ^7^ Department of Neurodegenerative Diseases and Geriatric Psychiatry, University Hospital Bonn, Bonn Germany; ^8^ Normandie Univ, UNICAEN, INSERM, U1237, PhIND “Physiopathology and Imaging of Neurological Disorders”, NeuroPresage Team, GIP Cyceron, Caen France; ^9^ Brigham and Women’s Hospital, Harvard Medical School, Boston, MA USA; ^10^ Center for Alzheimer’s Research and Treatment, Department of Neurology, Brigham and Women’s Hospital, Harvard Medical School, Boston, MA USA; ^11^ Amsterdam Neuroscience, Neurodegeneration, Amsterdam Netherlands; ^12^ Alzheimer Center Amsterdam, Department of Neurology, Vrije Universiteit Amsterdam, Amsterdam UMC, location VUmc, Amsterdam Netherlands; ^13^ Vrije Universiteit Amsterdam, Department of Clinical, Neuro‐ and Developmental Psychology, Amsterdam Netherlands; ^14^ Epidemiology and Data Science, Vrije Universiteit Amsterdam, Amsterdam UMC location VUmc, Amsterdam Netherlands; ^15^ Alzheimer Center Amsterdam, Neurology, Vrije Universiteit Amsterdam, Amsterdam UMC, Amsterdam Netherlands; ^16^ Faculty of Behavioural and Movement Sciences, Clinical Developmental Psychology & Clinical Neuropsychology, Vrije Universiteit Amsterdam, Amsterdam Netherlands; ^17^ Alzheimer’s disease and other cognitive disorders Unit. Hospital Clínic de Barcelona. Fundació de Recerca Clínic Barcelona – IDIBAPS. University of Barcelona, Barcelona Spain; ^18^ Centro de Investigación Biomédica en Red de Fragilidad y Envejecimiento Saludable (CIBERFES), Instituto de Salud Carlos III, Madrid Spain; ^19^ Barcelonaβeta Brain Research Center (BBRC), Barcelona Spain; ^20^ Hospital del Mar Research Institute (IMIM), Barcelona Spain; ^21^ Vanderbilt Memory & Alzheimer’s Center, Vanderbilt University Medical Center, Nashville, TN USA; ^22^ Wisconsin Alzheimer’s Disease Research Center, University of Wisconsin School of Medicine and Public Health, Madison, WI USA; ^23^ Division of Geriatrics, Department of Medicine, University of Wisconsin School of Medicine and Public Health, Madison, WI USA; ^24^ Melbourne School of Psychological Sciences, University of Melbourne, Melbourne, VIC Australia

## Abstract

**Background:**

In cognitively unimpaired (CU) older adults, the presence of a subjective cognitive decline (SCD) combined with evidence of abnormal b‐amyloid (Ab) is proposed as stage 2 of Alzheimer’s disease (AD) by the NIA‐AA framework (Jack et al., 2018). However, the associations found between SCD and preclinical AD are inconsistent across studies, highlighting the importance of better understanding which specific SCD features are associated with either Ab or tau burden.

**Methods:**

The present study includes cross‐sectional data from 9 independent cohorts with a total of 7217 CU older adults (57% female), aged 69.34 (1.20) years, recruited from general and memory‐clinic populations. Ab and tau biomarkers were measured by positron emission tomography (PET) or cerebrospinal fluid (CSF). Using established cut‐offs, 28% of participants were Aβ+, and 12% were Aβ+T+ (approximately one‐third of the sample had available tau data). We examined four SCD‐*plus* criteria as well as the mean number of SCD criteria met (i.e., mean SCD‐severity) in relation to both biomarker status and levels in logistic/linear regressions adjusted for age and sex for each cohort. Summary statistics were extracted for meta‐analyses.

**Results:**

The overall frequency of stage 2 AD varied from 7‐16% [4‐26%] according to each SCD‐*plus* criterion endorsement. Only 1‐5% [0‐8%] of participants meeting the SCD‐*plus* criteria also had both high Aβ and tau burden **(Fig.1)**. The presence of self‐reported memory decline (SMD), an associated concern/worry, and a higher mean SCD‐severity were each associated with high Ab (status and continuous). Only the latter was associated with high tau status **(Fig.2)**. Onset of SCD within the last 5 years, and feeling of worse performance than same‐age peers, were not associated with AD biomarkers at a Bonferroni‐corrected threshold.

**Conclusions:**

Our results suggest that widespread endorsement of multiple SCD features is more powerful than a single criterion alone in identifying both Aβ and tau in CU older adults. We found that the isolated SCD criterion was particularly sensitive to elevated Aβ, even after adjustment for tau, supporting the power of SCD as a very early behavioral marker of preclinical AD.